# Supplementary material for: In-Liquid Plasma Process for Size- and Shape-Controlled Synthesis of Silver Nanoparticles by Controlling Gas Bubbles in Water
Source: Materials (Basel). 2018 May 25;11(6):891. doi: 10.3390/ma11060891 (PMC6025040; doi:10.3390/ma11060891)
Supplement: Supplementary file 1 [file materials-11-00891-s001.zip › supplementary file S1 S2 S3.docx]

Supporting Information

In liquid plasma process process for size- and shape-controlled synthesis of silver nanoparticles by controlling gas bubbles in water

Hyun-Jin Kim Jun-Goo Shin, Choon-Sang Park, Dae Sub Kum, Bhum Jae Shin, Jae Young Kim, Hyung-Dal Park, and Heung-Sik Tae


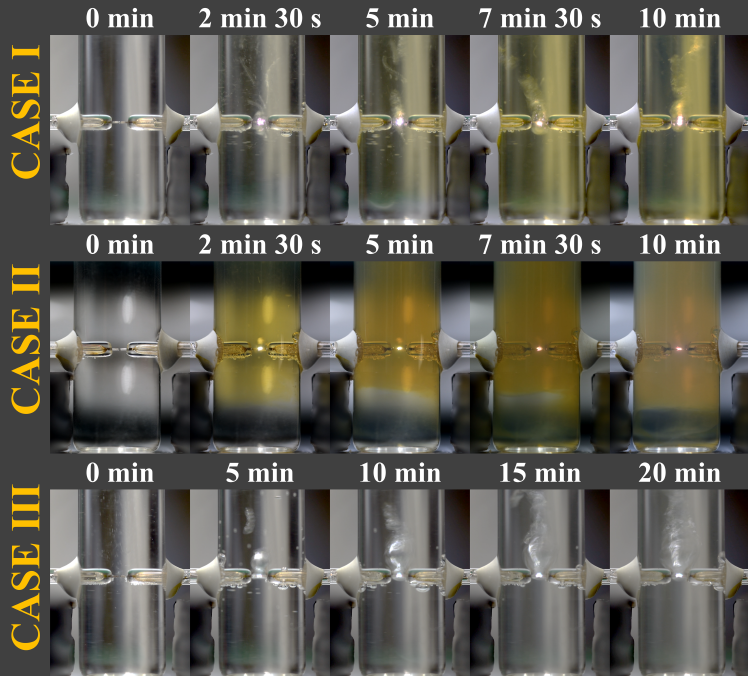


**Figure S1.** Shape of Ag electrodes after and before SPP treatment


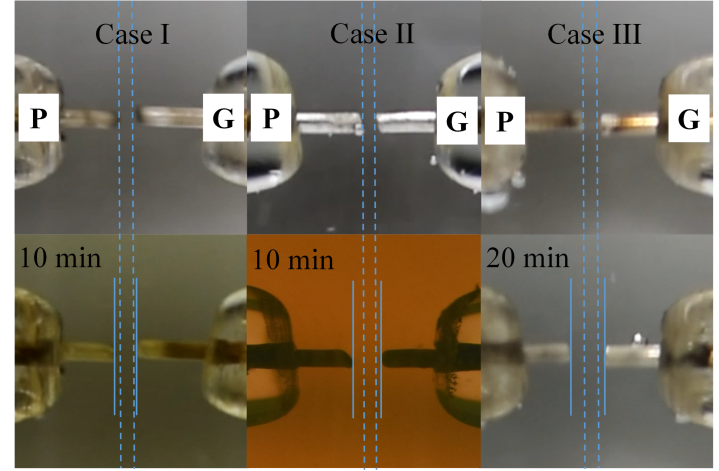


**Figure S2.** Change in length of powered and grounded Ag electrodes


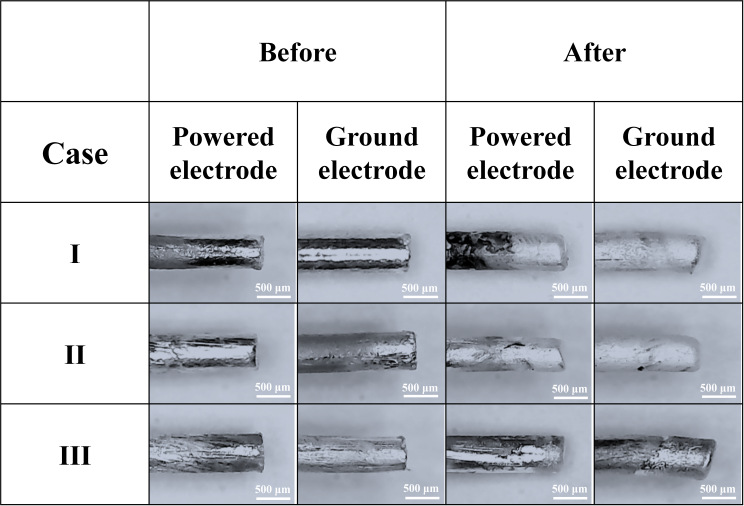


**Figure S3.** Shape of Ag electrodes after and before SPP treatment
