# Supplementary material for: In-Liquid Plasma Process for Size- and Shape-Controlled Synthesis of Silver Nanoparticles by Controlling Gas Bubbles in Water
Source: Materials (Basel). 2018 May 25;11(6):891. doi: 10.3390/ma11060891 (PMC6025040; doi:10.3390/ma11060891)
Supplement: Supplementary file 1 [file materials-11-00891-s001.zip › Finally Revised MS without Track Changes_materials-301951.docx]

Article

In-Liquid Plasma Process for Size- and Shape-Controlled Synthesis of Silver Nanoparticles by Controlling Gas Bubbles in Water

Hyun-Jin Kim ^1,2,†^, Jun-Goo Shin ^1,†^, Choon-Sang Park ^1^, Dae Sub Kum ^1^, Bhum Jae Shin ^3^,
Jae Young Kim ^4^, Hyung-Dal Park ^5^, Muhan Choi ^1^ and Heung-Sik Tae ^1,^*

^1^	School of Electronics Engineering, College of IT Engineering, Kyungpook National University, Daegu 41566, Korea; searchdre@ee.knu.ac.kr (H.-J.K.); bmw345@ee.knu.ac.kr (J.-G.S.);
purplepcs@ee.knu.ac.kr (C.-S.P.); kds890317@ee.knu.ac.kr (D.S.K.); mhchoi@ee.knu.ac.kr (M.C.)

^2^	SEMES Co. Ltd., Cheonan 31040, Chungcheongnam-do, Korea

^3^	Department of Electronics Engineering, Sejong University, Seoul 05006, Korea;
hahusbi@sejong.ac.kr

^4^	Department of New Biology, Daegu Gyeongbuk Institute of Science & Technology, Daegu 42988, Korea; jyk@dgist.ac.kr

^5^	Department of Mechanical Equipment Development, Radiation Technology eXcellence, Daejeon 34025, Korea; park.hyungdal@gmail.com

*****	Correspondence: hstae@ee.knu.ac.kr; Tel.: +82-53-950-6563

†	Hyun-Jin Kim and Jun-Goo Shin contributed equally to this work.

Received: 24 April 2018; Accepted: 24 May 2018; Published: date

**Abstract:** Most methods controlling size and shape of metal nanoparticles are chemical methods, and little work has been done using only plasma methods. Size- and shape-controlled synthesis of silver nanoparticles (Ag NPs) is proposed based on adjusting the gas bubble formation produced between two silver electrodes. The application of a voltage waveform with three different pulse widths during a plasma process in water can generate different gas bubble formations. Transmission electron microscopy (TEM) and scanning electron microscopy (SEM) images of Ag NPs synthesized using three different bubble formations reveal that spherical Ag NPs are synthesized when very tiny bubbles are generated between two electrodes or when only the grounded electrode is enveloped with large gas bubbles, but Ag nanoplates are synthesized when both electrodes are completely enveloped with large gas bubbles.

**Keywords:** controlled synthesis; gas bubble formation; voltage waveform; silver nanoparticle; silver nanoplate

1. Introduction

Solution (or liquid) plasma process (SPP, or LPP) and atmospheric pressure plasma (APP) are new useful and simple preparation methods of metal nanoparticles (NPs) because this nonequilibrium plasma can provide rapid reactions due to the reactive chemical species, i.e., radicals [1–5]. APP with low energy is affected by oxygen, nitrogen, and humidity in the atmosphere, as well as by its solvent and process gas [6]. However, SPP is only affected by solvent and process gas. Furthermore, in the case of SPP compared to APP method, high-energy radicals and a variety of reactive species are produced, thus resulting in directly synthesizing various NPs, such as metal [7–12], alloy [13–16], oxide [17–20], silicon [21,22], carbonaceous [23–25], and composites [26,27]. Metal NPs are applicable to various fields including electronics, catalysts, optoelectronics, imaging, sensing, and medical fields. When using metal NPs, precise control of the size and shape is required as the characteristics of metal NPs vary greatly depending on their size and shape. Thus, many methods for synthesizing diverse sizes and shapes have already been developed, especially using chemical solutions. However, such methods involve complex processes and high costs [4,5,28]. However, synthesizing diverse sizes and shapes by using SPP and APP is very difficult because the plasma is easily affected by ambient conditions. Therefore, studies related to the sizes and shapes of NPs synthesized by SPP (or LPP) and APP remain in the early stage compared to other synthesis methods.

It is generally considered that electrical breakdown in liquid occurs via the formation of bubbles [26,27]. As liquid plasma has various phases: liquid, gas (i.e., bubble) and plasma phases, the density of the plasma and reactive species during LPP can vary according to whether the discharge space consists of a gas–plasma interface or liquid–plasma. Thus, NPs synthesized using LPP require precise control of plasma parameters to obtain the desired NP properties and functionalities. However, controlling the size and shape of nanomaterials synthesized using LPP is very difficult as the discharge characteristics of liquid plasma are very sensitive to variations of gas bubble formations. This means that it would be not possible to control the size and shape of nanoparticles especially in the case of the in-liquid plasma process without precisely adjusting the gas bubble formations.

Gas bubbles in water are basically formed by vaporization due to Joule heating of the conduction current, implying that gas bubble formations can be significantly affected by adjusting the electrical pulsing methods, including voltage amplitude, while maintaining the other parameters, such as the boiling point, specific heat, conductivity, and liquid temperature [28–30]. That is, our idea is that the bubble formations can be controlled by adjusting the electrical pulsing methods, thereby resulting in enabling parameter control of the in-liquid plasma process for size- and shape-controlled synthesis of silver nanoparticles (Ag NPs).

Accordingly, this study monitored the bubble formation variation when adjusting the pulse width of the applied voltage waveform, and then investigated the resultant electrical and optical characteristics of plasma in deionized (DI) water depending on the bubble formation. As a result, Ag NPs with different sizes and shapes were synthesized depending on the bubble formation. Moreover, the experimental results confirmed the possibility of controlling the size and shape of synthesized Ag NPs by adjusting the gas bubble formation produced between two silver electrodes.

2. Materials and Methods

2.1. Liquid Plasma Reactor and Measurement Setup

A cylindrical glass tube (O.D. = 20 mm and I.D. = 18 mm) with a height of 150 mm was used as the plasma reactor. Ultrapure water (DI water: Welgene Inc., Gyeongsan-si, Korea, resistivity: 18 M Ω∙cm) was also used. To synthesize Ag NPs using LPP, two Ag wires (Nilaco Co. Ltd., Tokyo, Japan) with a diameter of 0.5 mm, covered with polytetrafluoroethylene (PTFE) and a glass tube, were used as the electrodes, one on each side of the plasma reactor. The gap between the electrodes was set at 1 mm, and only the tip of each electrode, up to 1 mm, was immersed in the DI water. Bipolar pulses with an amplitude of 3.8 kV and frequency of 5 kHz were applied to the electrodes. The power source used to generate bipolar pulses with various pulse widths consisted of a high-voltage amplifier (20/20C-HS, Trek Inc., Lockport, NY, USA) and pulse generator (AFG-3102, Tektronix Inc., Beaverton, OR, USA).

2.2. Pulsing Method for Different Bubble Formations

Three different bipolar pulse waveforms with different pulse widths were applied to the powered electrode to create different bubble formations, where the on-times were 20 μs for case I, 40 μs for case II, and 60 μs for case III. Increasing the pulse width of the voltage waveform meant increasing the supply of electrical energy in the water. Furthermore, since a longer pulse width implied a shorter off-period, the ensuing initial discharge was more likely to encounter a gas bubble-dominant state instead of a liquid-dominant state. Thus, it was expected that increasing the pulse width would help to improve the bubble sustainability prior to an ensuing discharge during continuous discharges.

2.3. Voltage-Current Measurement

To investigate the electrical characteristics, a high voltage probe (P6015A, Tektronix Inc., Beaverton, OR, USA) was connected to the powered electrode, while a current probe (Pearson Elec. Inc., Palo Alto, CA, USA) was connected to the grounded electrode.

2.4. Optical Emission Spectroscopy

The interactions between the plasma and the Ag electrodes during the LPP was investigated using optical emission spectrometer (Ocean Optics Inc., Dunedin, FL, USA) to measure the optical emission spectra via an optical guide installed on one side of the cylindrical glass tube.

2.5. ICCD Camera

The temporal behavior of the bubble formations and optical emission intensities originating from the plasma were measured using an intensified charge-coupled device (ICCD) camera (PI-MAX 2, Princeton Instruments, Trenton, NJ, USA) with an exposure time ranging from 1 to 10 ms in a shutter mode. Specifically, the bubble formations were measured using an exposure time of 1 ms corresponding to 5 cycles when the visible backlight was turned on. Meanwhile, the optical emission intensities were measured using an exposure time of 10 ms corresponding to 50 cycles when the visible backlight was turned off.

2.6. High-Speed Camera

A high-speed camera (Phantom Miro C110, AMETEK, Wayne, NJ, USA) and lens (Nikon AF Nikkor 105 mm, 1:2.8 D, Nikon, Tokyo, Japan) were used at 1800 frames per second (256 × 512 resolution) with a 555.55 μs shutter time to record and estimate the take-off (formation) time of the gas bubbles relative to three different pulse widths.

2.7. Scanning Electron Microscopy

The Ag NP analysis was performed using field emission-scanning electron microscopy (FE-SEM: SU8220, Hitachi Korea Co. Ltd., Seoul, Korea). During the FE-SEM measurement, the suspension sample was dropped onto a Si wafer and the suspension evaporated.

2.8. Transmission Electron Microscopy

The high-resolution (HR) transmission electron microscopy (TEM) images and selected area electron diffraction (SAED) patterns were taken with a Titan G2 ChemiSTEM Cs Probe (FEI Company, Hillsboro, OR, USA) transmission electron microscope, operating at 200 kV. A TEM sample of Ag nanoparticles was prepared by dropping suspension on carbon-coated copper grids. Energy dispersive X-ray spectroscopy (EDS) (FEI Company, Hillsboro, OR, USA) was also employed to validate the element composition and element spatial distribution of synthesized Ag nanoparticles.

2.9. Dynamic Light Scattering

The dynamic light scattering (DLS, Otsuka Electronics Co. Ltd., Osaka, USA) was used for size distribution measurement of Ag nanoparticles synthesized in water for three different cases.

2.10. Ultraviolet-Visible Light Spectroscopy

The ultraviolet-visible light spectroscopy (UV-Vis, BioSpectrometer, Eppendorf AG, Hamburg, Germany) was used for analysis of component and amount of Ag nanoparticles synthesized in water for three different cases at maximum absorption wavelength.

3. Results and Discussion

3.1. Variations in Discharge Characteristics According to Bubble Formation

Figure 1 shows a schematic diagram of the liquid plasma reactor and measurement setup used in this study. Under these experimental conditions, as the formation of bubbles was difficult due to the low conduction current of DI water at room temperature, prior to initiating the LPP, the DI water was pre-heated to about 80 °C using a hot plate and then monitored using a thermometer. As a result, bubbles were easily formed from the DI water when applying the bipolar pulse. Furthermore, the bubble formations were changed depending on the width of the applied bipolar pulse, and the DI water temperature was observed to be increased above 90 °C due to plasma heating. LPP times were determined for the synthesis rate of Ag NPs relative to the pulse width. For cases I and II, the process times were 5 and 10 min, whereas, for case III, the process times were 10 and 20 min. In addition, two samples were prepared for each case to examine the color and turbidity of the suspensions, as well as the size and shape of the synthesized Ag NPs.

In a gas-discharge, priming particles can help initiate a discharge by reducing the ignition voltage [31]. That is, the presence of priming particles, such as remaining charged particles and radicals, prior to initiating a discharge, can reduce the breakdown voltage of a gas-discharge.

In a similar manner, when liquid plasma is produced, the types of bubble formation at the time of discharge initiation can determine the liquid plasma discharge characteristics. Thus, liquid plasma features can vary considerably depending on the bubble formation characteristics, such as the sizes and amount of bubbles between the two electrodes. Therefore, this study adjusted the bipolar pulse waveforms using different pulse widths to examine the effect on the bubble formation between the two electrodes.

**Figure 1.** Schematic diagram of liquid plasma reactor and measurement setup used in this study.

Figure 2a shows ICCD images of the bubble formations generated in the DI water when using three voltage waveforms with different pulse widths (case I: 20 μs, case II: 40 μs, and case III: 60 μs). As shown, case I generated lots of tiny bubbles between the two Ag electrodes. Case II generated larger bubbles than case I, yet the bubbles were only enveloped around the grounded electrode, which meant that the powered electrode was the only electrode exposed to the liquid water.

**Figure 2.** (**a**) intensified charge-coupled device (ICCD) images of bubbles; (**b**) detailed images of gas bubble formation times using high speed camera; and (**c**) ICCD plasma intensities during liquid plasma discharge when applying three different bipolar pulses in Figure 1 to powered electrodes (P), where G is grounded electrode.

Finally, case III generated the largest bubbles, which were enveloped around both electrodes. High-speed images of the gas bubble formations induced by the underwater pulsed discharge with different pulse widths are shown in Figure 2b. Movies of the bubble formation and corresponding bubble behavior were induced when adjusting the pulse width are included (Videos S1, S2, and S3). As shown, for case I, tiny bubbles were irregularly generated by Joule heating due to a high current spike between the two Ag electrodes [32,33]. While this limited the measurement of a precise gas bubble formation time, the average formation time of one tiny bubble was 11.11 ms, corresponding to 111.11 half-pulses. For case II, the formation of one bubble was captured in 48 frames, meaning that the time required to form one bubble was 26.66 ms, corresponding to 266.66 half-pulses. For case III with a pulse width of 60 μs, the formation of one bubble was captured in 100 frames, meaning that the time required to form one bubble was 55.55 ms, corresponding to 555.55 half-pulses. When applying the voltage, this cycle was repeated in the context of continuous liquid and bubble motion. In case III, the volume of bubbles also increased despite a decreased electric current [32,33].

The bubble formation time for case III was much longer than that for case II, indicating that larger bubbles were generated with a longer pulse width. Moreover, as the bubbles in case III had a longer take-off time, they also had a longer duration than the bubbles in cases I and II. Figure 2c shows ICCD images (shutter mode) of the plasma intensities during the liquid plasma discharge for the three different cases. As previously described, the bubble formation was changed according to the pulse width, which meant variations in the plasma discharge initiation conditions based on the changes in the discharge space induced by the liquid–gas mix conditions between the two electrodes. As shown in Figure 2c, in case I where both Ag electrodes were exposed to water, the breakdown voltage increased as the plasma was produced in the liquid-dominant space with lots of tiny bubbles between the two electrodes. In particular, the liquid plasma intensity in case I was very strong in the vicinity of both electrodes. In case II where only the powered electrode was exposed to water, a strong discharge was observed near the powered Ag electrode. Meanwhile, in case III where both electrodes were completely enveloped with gas bubbles so that neither electrode was exposed to water, the plasma intensity was observed to be weak. Thus, the experimental results of Figure 2 reveal that the liquid plasma characteristics strongly depend on the pattern of the gas bubble formation. That is, the liquid plasma intensity was found to be inversely proportional to the bubble size, so that the corresponding discharge intensity was weakened with an increase in the bubble size. Therefore, since the discharge features of the liquid plasma were significantly affected by the bubble formations, the synthesis of Ag NPs was also investigated based on the liquid plasma characteristics related to the three different bubble formations.

Figure 3a shows the electrical model used to describe the plasma discharge region, where R_i_ is the leakage resistance, and C_w_ and R_w_ are the capacitance and resistance, respectively, of the DI water that can vary according to the liquid state. Figure 3b shows the peak of breakdown voltage and corresponding discharge current (V_p_-I_p_) when applying the three different voltages with different pulse-on periods (20 μs for case I, 40 μs for case II, and 60 μs for case III) to the powered electrode.

As shown in Figure 3b, in case I, after the first initial high voltage and current peaks, successive breakdown voltage and discharge current spikes followed immediately during the negative and positive half-cycles. In case II, successive breakdown voltages and discharge current spikes were observed during the negative half-cycle, yet not during the positive half-cycle. Finally, in case III, no successive voltages and current spikes were observed during either the negative or positive half-cycles. In general, since the streamer is propagated in the direction of the cathode, the occurrence of discharge current spikes during streamer propagation can vary depending on whether the streamer is propagated through a gas bubble dominant or liquid-dominant space. In the current experiment, the grounded electrode acted as the cathode during the positive-half cycles, while the powered electrode acted as the cathode during the negative-half cycles. Thus, in case I, liquid water comprised of tiny gas bubbles dominated the discharge space, causing the propagating streamer to experience a streamer hopping phenomenon [34–36]. As a result, current spikes were observed during both positive- and negative-half cycles. These current spikes mainly occurred due to an electric field enhancement at the bubble–liquid interface, giving rise to nascent electrons that created an avalanche process in the bubbles [34]. However, in case II where the gas bubbles were only enveloped around the grounded electrode, the streamer hopping phenomenon only occurred when the streamer propagated towards the powered electrode during a negative-half cycle. Meanwhile, in case III where both electrodes were completely enveloped with larger bubbles, the discharge space was located within gas bubbles, which resulted in no current spikes during either positive- or negative-half cycles.

Figure 4 shows the optical emission spectra (OES) for the plasma produced with three different bubble formations using an exposure time of 200 ms. As shown, in case I, the emission lines from hydrogen radicals (H_α_ at 656.3 nm and H_β_ at 486.1 nm) and oxygen radicals (O at 777 and 840 nm), originating from the dissociation of H_2_O, showed strong peaks. This indicates the production of relatively high amounts of hydrogen and oxygen radicals, which implies that, in case I, the plasma is produced in the discharge space with high amounts of liquid H_2_O between the two electrodes. As shown in case II of Figure 4, the emission lines from hydrogen and oxygen radicals were decreased with an increase in the pulse width because the increased bubble size contributed to reducing the portion of liquid H_2_O in the space between the two electrodes during the plasma discharge. Finally, with a further increase in the pulse width of the applied voltage waveform in case III, the emission lines from hydrogen radicals and oxygen radicals were significantly weakened, indicating a considerable increase in the portion of bubbles in the space between the two electrodes during the plasma discharge. Moreover, the results in Figure 4 show that increasing the bubble sizes also increased the intensities of the emission lines at 328.06 and 338.28 nm, corresponding to neutral silver transitions, whereas the intensities of the emission lines at 520.91 nm and 546.55 nm, corresponding to neutral silver transitions, were only slightly changed.

**Figure 3.** (**a**) electrical model of liquid plasma region and (**b**) three voltage waveforms with related breakdown voltages and discharge currents (V_p_-I_p_) measured during one cycle with different on-times; 20 μs for case I, 40 μs for case II, and 60 μs for case III.

**Figure 4.** Optical emission spectra (OES) for plasma generated by three bubble formations shown in Figure 2 with exposure time of 200 ms.

3.2. Changes in Sizes and Shapes of Ag NPs According to Bubble Formations

Figure 5 shows SEM images of the Ag NPs synthesized in cases I, II, and III. While spherical Ag NPs were synthesized in cases I and II, large size polygonal Ag nanoplates were synthesized in case III, indicating that the synthesis mechanism varied according to the bubble formation. Plus, in all cases, the sizes of the Ag NPs were increased by increasing the process time. Figure 6 shows TEM images of Ag NPs synthesized by SPP (or LPP). The average size distributions of the Ag particle sizes in cases I, II, and III were found to be in the range from 13 to 37 nm in Figure 6a, from 36 to 53 nm in Figure 6b, form 0.2 to 0.87 µm in Figure 6c, respectively, which agreed well with the SEM results. As shown in Figure 6, when the pulse width was increased, Ag nanoparticles sizes were increased. The SAED patterns of Ag NPs in all cases revealed the clear diffraction rings and spots of the polycrystalline characteristics. The SAED patterns indicated that the lattice spacings in Figure 6a were 0.217 and 0.123 nm corresponding to (200) and (311) crystal planes of face-centered cubic (fcc) silver (International Centre for Diffraction Data (ICDD) card no. 03-065-2871), respectively. The lattice spacings in Figure 6b were 0.24 and 0.127 nm corresponding to (111) and (311) crystal planes of fcc silver, respectively. The lattice spacings in Figure 6c were 0.254, 0.149, and 0.099 nm corresponding to (111), (220), and (400) crystal planes of fcc silver, respectively [37–41]. The EDS patterns in the top-right insets of Figure 6a–c show that these particles are silver. Accordingly, the proposed method of SPP with the three different bubble formations induced by adjusting the pulse widths can illustrate the possibility of controlling the size and shape of Ag NPs.

**Figure 5.** Scanning electron spectroscopy (SEM) images of synthesized Ag nanoparticles (NPs) through solution plasma process (or liquid plasma process) SPP (or LPP) for three cases.

**Figure 6.** Transmission electron microscopy (TEM) images in (**a**) case I, (**b**) case II, and (**c**) case III. Top-right corner insets in (**a**–**c**) are energy dispersive X-ray spectroscopy (EDS). Bottom-left corner insets in (**a**–**c**) are selected area electron diffraction (SAED) patterns of synthesized Ag NPs for three cases.

Figure 7 shows the color variations of the Ag NP suspensions according to the process time for each case (Figure S1). For case I, the Ag NP suspension was changed from clear to light-yellow to yellowish-brown during the process time. For case II, the Ag NP suspension was changed from clear to yellowish-brown to milky after a 10 min process time. However, for case III, the Ag NP suspension remained clear throughout the 20 min process time.

**Figure 7.** Color changes of Ag NP suspensions for three different cases according to plasma process time.

Figure 8 shows the UV-Vis absorption spectra for six Ag NP suspension samples and corresponding colors of the Ag NP suspensions. The absorption spectra for silver spherical nanoparticles suspended in water are already known to be near 400 nm and their peaks are red-shifted with an increase in the sphere size, which is mainly due to the surface plasmon resonance (SPR) phenomenon [11,42].

**Figure 8.** UV-Vis absorption spectra for six different Ag NP suspensions prepared by LPP: (**a**) UV-Vis absorption spectra and ((**b**) inset) corresponding colors of Ag NP suspensions.

Figure 9 shows the results of dynamic light scattering (DLS) measurement for cases I and II, respectively. In case I, the sizes of Ag NPs were ranged from 25 to 130 nm at the process time of 5 min. When the process time was increased to 10 min, their sizes were increased from 25 to 200 nm. In case II, when the process time was 5 min, their sizes were ranged from 30 to 350 nm, and the size distribution was widened, as shown in Figure 9b. It is noted that the portion of the sizes larger than 100 nm is considerably increased when the process time is increased to 10 min, indicating that the uniformity of the size decreases while the size increases with an increase in the process time.

|  |  |
| --- | --- |
| (**a**) | (**b**) |

**Figure 9.** Dynamic light spectroscopy (DLS) measurement of synthesis Ag NPs (**a**) case I and (**b**) case II.

As shown by the SEM images in Figure 5 and TEM images in Figure 6, the shapes of the Ag NPs synthesized in cases I and II were spherical, which was also confirmed by the UV-Vis absorption peaks for cases I and II in Figure 8a. For cases I and II, the UV-Vis absorption peaks tended to be red-shifted with an increase in the process time. In case III, however, the shapes of the Ag NPs were plates, as shown by the SEM image in Figure 5 and TEM images in Figure 6, and, therefore, the UV-Vis absorption spectra in Figure 8a showed no peaks [33]. Plus, the Ag NP suspensions remained clear, as shown in Figure 8b.

Figure 10 shows microscope and SEM images of changes in the Ag electrode tips after 10 min LPP with the three different bubble formations induced by adjusting the pulse width. As shown in Figure 10a, in all cases, the Ag electrode lengths were reduced after treatment (Figures S2 and S3). Moreover, the powered Ag electrodes were more damaged than the grounded Ag electrodes probably because the streamer discharge plasma would cause the powered Ag electrode to be eroded or sputtered more severely due to the larger potential difference between the powered electrode and the liquid plasma medium. As shown in Figure 10b, in cases I and II, the Ag electrode was severely eroded by being melted down, where the thermal damage was due to the high current, resulting in the synthesis of spherical Ag NPs, as shown in Figure 5. Meanwhile, in case III, the Ag electrode showed a cracked surface, indicating both sputtering and melting induced by severe ion bombardment and thermal damage, respectively, as the Ag electrodes were completely enveloped by gas bubbles, as shown by the ICCD and bubble images in Figure 2. As a result, large-size polygonal Ag nanoplates were synthesized from the cracked surfaces of the Ag electrode in case III. In conclusion, the proposed method of SPP with the three different bubble formations induced by adjusting the pulse widths can easily control the shape and size of metal NPs. When the pulse width was decreased, small size spherical NPs were generated with lots of tiny bubbles between the two Ag electrodes due to the produced erosion of electrode caused by the intense plasma produced. However, when the pulse width was increased, large size polygonal nanoplates were generated with large bubbles due to produced sputtering and melting of electrode induced by severe ion bombardment and thermal damage, as the electrodes were completely enveloped by large gas bubbles.

**Figure 10.** (**a**) microscope and (**b**) SEM images of changes in Ag electrode tips exposed to discharges after 10-min LPP with three different bubble formations induced by adjusting pulse width.

4. Conclusions

When synthesizing Ag NPs using LPP, controlling the agent gas bubble generation between the two electrodes is very important. This study applied bipolar pulses with different pulse widths to Ag electrodes to produce three different bubble formations in the liquid plasma channel. The resulting changes in the electrical and optical emission characteristics of the plasmas generated according to the three different bubble formations were then examined using ICCD, a high-speed camera, OES, and V_p_-I_p_ measurement techniques. TEM and SEM images of the resulting Ag NPs revealed that spherical Ag NPs were synthesized either when very tiny bubbles were generated between two electrodes, or when large gas bubbles were enveloped around the grounded electrode, whereas Ag nanoplates were synthesized when both electrodes were completely enveloped with large gas bubbles. In conclusion, the gas bubble conditions were confirmed to play a significant role in the size-and shape-controlled synthesis of silver nanoparticles during LPP, and Ag nanoparticles with different sizes and shapes were successfully grown with different gas bubble formations induced by adjusting the pulse width of the bipolar voltage waveform.

**Supplementary Materials:** The following are available online at www.mdpi.com/xxx/s1, Figure S1: Solution color changes for three cases according to plasma process time, Figure S2: Change in length of powered and grounded Ag electrodes, Figure S3: Shape of Ag electrodes after and before SPP treatment, Video S1: bubble formation of 20 μs voltage pulse width, Video S2: bubble formation of 40 μs voltage pulse width, Video S3: bubble formation of 60 μs voltage pulse width.

**Acknowledgments:** This work was supported in part by a National Research Foundation of Korea (NRF) grant funded by the Korea government (MSIP) (No. 2017R1A4A1015565) and in part by Brain Korea 21 (BK21) plus project funded by the Ministry of Education, Korea (No. 21A20131600011).

**Author Contributions:** H.-J.K., J.-G.S., and H.-S.T. conceived and designed the study; H.-J.K., J.-G.S., C.-S.P., and D.S.K. performed the experiments; H.-D.P. help to consist of plasma experimental setup; H.-J.K., J.-G.S., D.S.K., B.J.S., J.Y.K., H.-D.P., C.-S.P., M.C., and H.-S.T. analyzed the data; H.-J.K., J.-G.S., C.-S.P., and H.-S.T. wrote the majority of the paper and all authors reviewed and approved the final version.

**Funding:** This research was funded in part by a National Research Foundation of Korea (NRF) grant funded by the Korea government (MSIP) (No. 2017R1A4A1015565) and in part by Brain Korea 21 (BK21) plus project funded by the Ministry of Education, Korea (No. 21A20131600011).

**Conflicts of Interest:** The authors declare no conflict of interest.

References

1. Yoshida, T.; Yamamoto, N.; Mizutani, T.; Yamamoto, M.; Ogawa, S.; Yagi, S.; Nameki, H.; Yoshida, H. Synthesis of Ag nanoparticles prepared by a solution plasma method and application as a cocatalyst for photocatalytic reduction of carbon dioxide with water. *Catal. Today* **2018**, *303*, 320–326. doi:10.1016/j.cattod.2017.08.047.
2. Vos, C.D.; Baneton, J.; Witzke, M.; Dille, J.; Godet, S.; Gordon, M.J.; Sankaran, R.M.; Reniers, F. A comparative study of the reduction of silver and gold salts in water by a cathodic microplasma electrode. *J. Phys. D Appl. Phys.* **2017**, *50*, 105206. doi:10.1088/1361-6463/aa5608.
3. Heo, Y.K.; Kim, S.M.; Lee, S.Y. Effects of discharge duration on the size and shape of gold nanoparticles synthesized using solution plasma processing. *Phys. Scr.* **2010**, *T139*, 014025. doi.10.1088/0031-8949/2010/T139/014025.
4. Hieda, J.; Saito, N.; Takai, O. Exotic shapes of gold nanoparticles synthesized using plasma in aqueous solution. *J. Vac. Sci. Technol. A* **2008**, *26*, 854–856. doi.org/10.1116/1.2919139.
5. Bratescu, M.A.; Cho, S.P.; Takai, O.; Saito, N. Size controlled gold nanoparticles synthesized in solution plasma. *J. Phys. Chem. C* **2011**, *115*, 24569–24576, doi:10.1021/jp207447c.
6. Mun, M.K.; Lee, W.O.; Park, J.W.; Kim, D.S.; Yeom, G.Y.; Kim, D.W. Nanoparticle synthesis and modification using solution plasma process. *Appl. Sci. Converg. Technol.* **2016**, *26*, 164–173, doi:10.5757/ASCT.2017.26.6.164.
7. Saito, G.; Akiyama, T. Nanomaterial synthesis using plasma generation in liquid*. J. Nanomater*. **2015**, *2015*, 123696. doi:10.1155/2015/123696.
8. Watthanaphanit, A.; Panomsuwan, G.; Saito, N. A novel one-step synthesis of gold nanoparticles in an alginate gel matrix by solution plasma sputtering. *RSC Adv*. **2014**, *4*, 1622–1629, doi:10.1039/C3RA45029E.
9. Jin, S.H.; Kim, S.M.; Lee, S.Y.; Kim, J.W. Synthesis and characterization of silver nanomaterials using solution plasma process. *J. Nanosci. Nanotechnol*. **2014**, *14*, 8904–8097, doi:0.1166/jnn.2014.9428.
10. Mizutani, T.; Ogawa, S.; Murai, T.; Nameki, H.; Yoshida, T.; Yagi, S. In situ UV–vis investigation of growth of gold nanoparticles prepared by solution plasma sputtering in NaCl solution. *Appl. Surf. Sci.* **2015**, *354*, 397–400, doi:10.1016/j.apsusc.2015.04.139.
11. Pootawang, P.; Saito, N.; Takai, O. Synthesis and characteristics of Ag, Pt bimetallic nanocomposites by arc-discharge solution plasma processing. *Nanotechnology* **2012**, *23*, 395602, doi:10.1088/0957-4484/23/39/395602.
12. Hu, X.; Shen, X.; Takai, O.; Saito, N. Facile fabrication of PtAu alloy clusters using solution plasma sputtering. *J. Alloys Compd*. **2013**, *552*, 351–355, doi:10.1016/j.jallcom.2012.08.033.
13. Kim, S.M.; Lee, Y.J.; Kim, J.W.; Lee, S.Y. Facile synthesis of Pt–Pd bimetallic nanoparticles by plasma discharge in liquid and their electrocatalytic activity toward methanol oxidation in alkaline media. *Thin Solid Films* **2014**, *572*, 260–265, doi:10.1016/j.tsf.2014.07.067.
14. Lee, S.-J.; Lee, H.; Jeon, K.-J.; Park, H.; Park, Y.-K.; Jung, S.-C. Characterization of Bimetallic Fe-Ru Oxide nanoparticles prepared by liquids-phase plasma method. *Nanoscale Res. Lett*. **2016**, *11*, 344, doi:10.1186/s11671-016-1557-8.
15. Saito, G.; Hosokai, S.; Tsubota, M.; Akiyama, T. Influence of Solution Temperature and Surfactants on Morphologies of Tin Oxide Produced Using a Solution Plasma Technique. *Cryst. Growth Des*. **2012**, *12*, 2455–2459, doi:10.1021/cg300083k.
16. Chen, Q.; Kaneko, T.; Hatakeyama, R. Synthesis of Superfine Ethanol-Soluble CoO Nanoparticles via Discharge Plasma in Liquid. *Appl. Phys. Express* **2012**, *5*, 96201, doi:10.1143/APEX.5.096201.
17. Nakasugi, Y.; Saito, G.; Yamashita, T.; Akiyama, T. Synthesis of nonstoichiometric titanium oxide nanoparticles using discharge in HCL solution. *J. Appl. Phys*. **2014**, *115*, 123303, doi:10.1063/1.4869126.
18. Kelgenbaeva, Z.; Omurzak, E.; Ihara, H.; Iwamoto, C.; Sulaimankulova, S.; Mashimo, T. Sn and SnO_2_ nanoparticles by pulsed plasma in liquid: Synthesis, characterization and applications. *Phys. Status Solidi A* **2015**, *212*, 2951–2957, doi:10.1002/pssa.201532502.
19. Askari, S.; Macias-Montero, M.; Velusamy, T.; Maguire, P.; Svrcek, V.; Mariotti, D. Silicon-based quantum dots: Synthesis, surface and cmposition tuning with atmospheric pressure plasma. *J. Phys. D Appl. Phys*. **2015**, *48*, 314002, doi:10.1088/0022-3727/48/31/314002.
20. Wu, J.J.; Kondeti, V.S.S.K.; Bruggeman, P.J.; Kortshagen, U.R. Luminescent, water-soluble silicon quantum dots via micro-plasma surface treatment. *J. Phys. D Appl. Phys*. **2016**, *49*, doi:10.1088/0022-3727/49/8/08LT02.
21. Omurzak, E.; Abdullaeva, Z.; Iwamoto, C.; Ihara, H.; Sulaimankulova, S.; Mashimo, T. Synthesis of Hollow Carbon Nano-Onions Using the Pulsed Plasma in Liquid. *J. Nanosci. Nanotechnol*. **2015**, *15*, 3703–3709, doi:10.1166/jnn.2015.9278.
22. Kim, S.H., Dao, V.D.; Larina, L.L.; Jung, K.D.; Choi, H.S. Solution-processable rGO–Pt nanohybrids synthesized in an aqueous fructose solution for transparent and efficient dye-sensitized solar cells. *Chem. Eng. J.* **2016**, *283*, 1285–1294, doi:10.1016/j.cej.2015.08.070.
23. Morishita, T.; Ueno, T.; Panomsuwan, G.; Hieda, J.; Yoshida, A.; Bratescu, M.A.; Saito, N. Fastest Formation Routes of nanocarbons in solution plasma processes. *Sci. Rep*. **2016**, *6*, 36880, doi:10.1038/srep36880.
24. Abdullaeva, Z.; Omurzak, E.; Iwamoto, C.; Ihara, H.; Ganapathy, H.S.; Sulaimankulova, S.; Koinuma, M.; Mashimo, T. Pulsed Plasma Synthesis of Iron and Nickel Nanoparticles Coated by Carbon for Medical applications. *Jpn. J. Appl. Phys*. **2013**, *52*, doi:10.7567/JJAP.52.01AJ01.
25. Lee, Y.J.; Kim, S.M.; Kim, J.W.; Lee, S.Y.; The characterization and electrocatalytic activities of carbon-supported Pt nanoparticles synthesized by the solution plasma process. *Mater. Lett*. **2014**, *123*, 184–186, doi:10.1016/j.matlet.2014.03.022.
26. Chen, Q.; Li, J.; Li, Y. A reviews of plasma-liquid interactions for nanomaterial synthesis. *J. Phys. D Appl. Phys*. **2015**, *48*, 424005, doi:10.1088/0022-3727/48/42/424005.
27. Bruggeman, P.; Leys, C. Non-thermal plasmas in and in contact with liquids. *J. Phys. D Appl. Phys*. **2009**, *42*, 53001, doi:10.1088/0022-3727/42/5/053001.
28. Wang, F.; Tang, R.; Yu, H.; Gibbons, P.C.; Bruhro, W.E. Size and shape controlled synthesis of bismuth nanoparticle. *Chem. Mater.* **2008**, *20*, 3656–3662, doi:10.1021/cm8004425.
29. Hsieh, A.; Chang, H.; Hsu, C. The bubble to jetting transition mechanism of plasmas in NaNO_3_ solutions sustained by pulsed power. *J. Phys. D Appl. Phys.* **2012**, *45*, 415202, doi:10.1088/0022-3727/45/41/415202.
30. Chang, H.W.; Hsu, C.C. Plasmas in Saline Solution Sustained Using Bipolar Pulsed Power Source: Tailoring the Discharge Behavior Using the Negative Pulses. *Plasma Chem. Plasma Process*. **2013**, *33*, 581–591, doi:10.1007/s11090-013-9447-7.
31. Sobel, A. Gas-discharge displays: The state of the art. *IEEE Trans. Electron Devices* **1977**, *24*, 835–847, doi:10.1109/T-ED.1977.18839.
32. Uehara, S.; Ishihara, K.; Nishiyama, H. Development of a capillary plasma pump with vapor bubble for water purification: Experimental and theoretical investigation. *J. Phys. D Appl. Phys.* **2016**, *49*, 405202, doi:10.1088/0022-3727/49/40/405202.
33. Zhang, L.; Zhu, X.; Yan, H.; Huang, Y.; Liu, Z.; Yan, K. Synthesis of SnO_2_ nanoparticles using a solution plasma and their gas-sensing properties. *Appl. Phys. Lett*. **2017**, *110*, doi:10.7567/JJAP.55.01AE17.
34. Qian, J.; Joshi, R.P.; Kolb, J.; Schoenbach, K.H.; Dickens, J.; Neuber, A.; Butcher, M.; Cevallos, M.; Krompholz, H.; Schamiloglu, E.; et al. Microbubble-based model analysis of liquid breakdown initiation by a submicrosecond pulse. *J. Appl. Phys*. **2005**, *97*, 113304, doi:10.1063/1.1921338.
35. Sommers, B.S.; Foster, J.E.; Nonlinear oscillations of gas bubbles submerged in water: Implications for plasma breakdown. *J. Phys. D Appl. Phys*. **2012**, *45*, 415203, doi:10.1088/0022-3727/45/41/415203.
36. Foster, J.E. Plasma-based water purification: Challenges and prospects for the future. *Phys. Plasmas* **2017**, *24*, 055501, doi:10.1063/1.4977921.
37. Vinod, M.; Gopchandran, K.G. Au, Ag and Au:Ag colloida nanoparticles synthesized by pulsed laser ablations as SERS substrates. *Pro. Nat. Sci. Mater.* **2014**, *24*, 569–578, doi:10.1016/j.pnsc.2014.10.003.
38. He, X.; Zhao, X.; Li, Y.; Sui, X. shape-controlled synthesis for silver: Trangular/hexagonal nanoplates, chain-like nanoplate assemblies, and nanobelts. *J. Mater. Res.* **2009**, *24*, 2200–2209, doi:10.1557/JMR.2009.0264.
39. Xin, J.; Yin, X.; Chen, S.; Wu, A. Synthesis of uniform and stable silver nanoparticles by a gold seed-mediated growth approach in a buffer system. *J. Exp. Nanosci*. **2012**, *9*, 382–390, doi:10.1080/17458080.2012.662656.
40. Park, C.-S.; Kim, D.H.; Shin, B.J.; Kim, D.Y.; Lee, H.-K.; Tae, H.-S. Conductive polymer synthesis with single-crystallinity via a novel plasma polymerization technique for gas sensor applications. *Materials* **2016**, *9*, 812, doi:10.3390/ma9100812.
41. Kim, D.H.; Park, C.-S.; Kim, W.H.; Shin, B.J.; Hong, J.G.; Park, T.S.; Seo, J.H.; Tae, H.-S. Influence of guide-tube and bluff-body on advanced atmospheric pressure plasma source for single-crystlline polymer nanoparticle synthesis at low temperature. *Phys. Plasmas* **2017**, *24*, 023505, doi:10.1063/1.4975313.
42. Cobley, C.M.; Skrabalak, S.E.; Campbell, D.J.; Xia, Y. Shape-controlled synthesis of silver nanoparticles for plasmonic and sensing applications. *Plasmonics* **2009**, *4*, 171–179, doi:10.1007/s11468-009-9088-0.

© 2018 by the authors. Submitted for possible open access publication under the
terms and conditions of the Creative Commons Attribution (CC BY) license (http://creativecommons.org/licenses/by/4.0/).
